# Ligand binding to a G protein–coupled receptor captured in a mass spectrometer

**DOI:** 10.1126/sciadv.1701016

**Published:** 2017-06-16

**Authors:** Hsin-Yung Yen, Jonathan T. S. Hopper, Idlir Liko, Timothy M. Allison, Ya Zhu, Dejian Wang, Monika Stegmann, Shabaz Mohammed, Beili Wu, Carol V. Robinson

**Affiliations:** 1 Chemistry Research Laboratory, University of Oxford, South Parks Road, Oxford OX1 3QZ, UK.; 2 OMass Technologies Ltd., Centre for Innovation and Enterprise, Begbroke Science Park, Woodstock Road, Oxford OX5 1PF, UK.; 3 CAS Key Laboratory of Receptor Research, Shanghai Institute of Materia Medica, Chinese Academy of Sciences, 555 Zuchongzhi Road, Pudong, Shanghai 201203, China.; 4 School of Life Science and Technology, ShanghaiTech University, 99 Haike Road, Pudong, Shanghai 201203, China.; 5 Departments of Chemistry and Biochemistry, University of Oxford, Oxford OX1 3QU, UK.

**Keywords:** G-protein coupled receptors, mass spectrometry, ligand binding

## Abstract

G protein (heterotrimeric guanine nucleotide–binding protein)–coupled receptors belong to the largest family of membrane-embedded cell surface proteins and are involved in a diverse array of physiological processes. Despite progress in the mass spectrometry of membrane protein complexes, G protein–coupled receptors have remained intractable because of their low yield and instability after extraction from cell membranes. We established conditions in the mass spectrometer that preserve noncovalent ligand binding to the human purinergic receptor P2Y_1_. Results established differing affinities for nucleotides and the drug MRS2500 and link antagonist binding with the absence of receptor phosphorylation. Overall, therefore, our results are consistent with drug binding, preventing the conformational changes that facilitate downstream signaling. More generally, we highlight opportunities for mass spectrometry to probe effects of ligand binding on G protein–coupled receptors.

## Teaser

Drug binding to the P2Y_1_ receptor prevents phosphorylation and competes with an endogenous ligand.

## INTRODUCTION

G protein (heterotrimeric guanine nucleotide–binding protein)–coupled receptors (GPCRs) are the largest family of membrane proteins in vertebrates and account for nearly 40% of targets for current drugs (*1*). The entire GPCR family has ~1000 members that share a conserved topology with seven transmembrane helices and are categorized into six classes according to structural and functional criteria (*2*, *3*). GPCR activation is thought to involve rearrangement of the transmembrane helices induced by ligand binding, one of the critical steps that triggers activation for downstream signaling with G proteins and modulation of cellular physiology (*4*, *5*). Agonist-induced conformational changes of GPCRs also expose sites on the intracellular regions that can be modified by phosphorylation (*6*). This combined process of conformational change and posttranslational modification (PTM) allows the receptor to interact with a number of GPCR-interacting proteins to desensitize GPCRs (*7*, *8*), the arrestin family being the most extensively studied.

The human purinergic receptor P2Y_1_R, a class A GPCR, functions as a receptor for extracellular adenosine 5′-diphosphate (ADP) and adenosine 5′-triphosphate (ATP) and regulates many physiological processes including thrombosis (*9*–*13*). P2Y_1_R is fully activated by ADP to facilitate platelet aggregation via calcium wave propagation. Inhibition of P2Y_1_R activation leads to a significant decrease in ADP-induced platelet aggregation (*14*). Thus, P2Y_1_R acts as a key antithrombotic drug target (*10*). MRS2500 {(1′*R*,2′*S*,4′*S*,5′*S*)-4-(2-iodo-6-methylamino-purine-9-yl)-1-[(phosphato)methyl]-2-(phosphato)bicyclo[3.1.0]-hexane} is a potent antagonist of P2Y_1_R, inhibiting ADP-platelet aggregation and reducing arterial thrombosis (*15*–*17*). X-ray crystal structures of ligand-bound P2Y_1_R and mutagenesis studies reveal that binding to MRS2500 involves the same pocket responsible for ADP binding. MRS2500, in contrast to ADP binding, stabilizes the receptor in an inactive conformation by reducing the conformational flexibility of helices VI and VII, resulting in a severe loss of receptor activity (*18*).

Nondenaturing or native mass spectrometry (MS) has emerged as a powerful technology for studying intact membrane protein assemblies (*19*). Modified Orbitrap MS can provide the resolving power necessary to define noncovalent interactions of membrane proteins with small molecules, as well as to identify protein modifications, including glycosylation and phosphorylation (*20*–*22*). To date, although excellent MS data have been recorded for large membrane complexes including intact rotary adenosine triphosphatases (*23*) and for peptides derived from GPCRs following chemical cross-linking or hydrogen deuterium exchange (*24*, *25*), it has been challenging to study intact GPCRs with ligand binding maintained. We attribute this challenge to the instability of GPCRs when removed from their native membranes in micelles, a property that is further compounded by their low expression yield and the requirement to remove the micelle in the mass spectrometer by activation in the gas phase.

## RESULTS

**Fig. 1:**
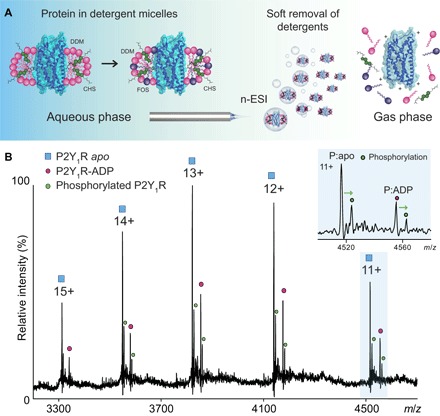
Schematic representation of the experiment designed to preserve the folded structure of the GPCR during transfer from solution to gas phase and resulting mass spectra, revealing binding of an endogenous ligand. (**A**) Mixed micelles composed of cholesterol and detergent are formed in solution encapsulating P2Y_1_R. Gas phase activation is used to release the GPCR, effecting transfer from solution to gas phase while retaining the ligand-binding site. nESI, nanoelectrospray ionization. (**B**) MS of wild-type P2Y_1_R reveals two peaks for each charge state corresponding to apo (blue square) and binding of endogenous ADP (magenta circle). Expansion of the 11+ charge state (blue background) shows significant phosphorylation (green) of apo and ligand-bound forms. *m*/*z*, mass/charge ratio.

After extensive detergent screening, we were able to optimize solution and MS conditions to enable preservation of folded ligand-bound human P2Y_1_R. The receptor, which was purified from insect cells as described previously (*18*), was introduced into the gas phase from a mixed detergent micelle, containing *n*-dodecyl β-d-maltoside (DDM), cholesterol, and foscholine (Fig. 1A; see Materials and Methods). This micelle was then destabilized in the gas phase, through collisions with neutral gas molecules, enabling us to obtain mass spectra of the naked receptor and consequently define the mass of P2Y_1_R in its apo state (table S1). In the mass spectrum, in addition to the series assigned to the apo protein, a predominant adduct peak was observed with a mass increase of 426.7 ± 2 Da, corresponding in mass to noncovalent binding of ADP (Fig. 1B). We did not detect ATP binding to the receptor, although ATP was added during the early stages of the purification (see Materials and Methods). We conclude that ATP is hydrolyzed such that ADP is retained as the endogenous ligand of P2Y_1_R. Observation of ADP binding implies that the ligand-binding pocket of the receptor is preserved in the gas phase. We also observed significant phosphorylation of the receptor, enabling us to explore the effects of this modification on ADP binding. We noted that the ratio of the peak intensities assigned to the phosphorylated forms of apo and ADP-bound receptor is indistinguishable within error (39.6 and 41.5%), as determined by UniDec (Universal Deconvolution) software (*26*). This correspondence implies that the phosphorylation process is not affected by the binding of ADP/ATP to P2Y_1_R.

**Fig. 2:**
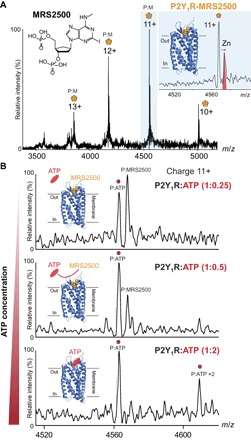
Mass spectrum of P2Y_1_R isolated with drug and incubated with different molar ratios of ATP. (**A**) Mass spectrum of P2Y_1_R without ATP incubation shows 100% binding to the drug, with no evidence for phosphorylation. (**B**) Mass spectra recorded following addition of ATP at a 1:0.25 molar ratio of protein to ADP show displacement of the drug by ATP. Stepwise increase of ATP to P2Y_1_R from 1:0.5 and 1:2 molar ratios shows further displacement of the drug by ATP and also evidence of a second putative binding site for ATP (bottom).

To establish whether drug binding to P2Y_1_R could be preserved within the gas phase, we purified the receptor in the presence of MRS2500 (*18*). Under MS conditions similar to those used for the apo protein, we observed a single charge state distribution corresponding to the theoretical mass of the receptor-drug complex (Fig. 2 and table S1). The apo protein was not observed in the mass spectrum, implying that 100% drug binding is maintained throughout purification and during transfer into the gas phase. High-energy collisions, designed to activate the complex, led to dissociation of MRS2500, likely through unfolding of the GPCR (fig. S1). We also observed a significant population of zinc binding that was assigned to the rubredoxin fusion protein used for x-ray crystallography of P2Y_1_R-MRS2500 (*18*). In the presence of the drug, we could not detect any of the phosphorylated form of P2Y_1_R, indicating that MRS2500 binding inhibits phosphorylation (*6*). Our mass spectra of the receptor-drug complex also allow us to rule out simultaneous binding to endogenous ADP, thereby confirming the higher affinity of P2Y_1_R for MRS2500 over ADP (*15*, *17*).

Given our observation of endogenous nucleotide binding, we examined the ability of different molar ratios of exogenous ATP to compete with MRS2500 (Fig. 2). We incubated the receptor-drug complex at 37°C for 30 min with 0.25 and 0.5 molar ratios of ATP and found that both ATP and drug-bound P2Y_1_R are observed with these low concentrations. Incubation at higher ATP concentrations (1:2 molar P2Y_1_R/ATP) raised the possibility of a second ATP-binding site and demonstrated complete displacement of MRS2500 by substoichiometric quantities of ATP.

**Fig. 3:**
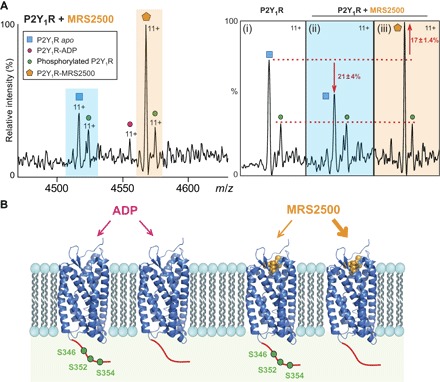
Comparison of mass spectra recorded for MRS2500 binding to phosphorylated and unmodified forms of P2Y_1_R. (**A**) Mass spectrum recorded following addition of MRS2500 to P2Y_1_R assigned as follows: unmodified P2Y_1_R (blue background/square) with phosphorylation (green circle), endogenous ADP binding (red circle), and MRS2500 binding to P2Y_1_R (orange background/hexagon). (i) Comparison of the peak heights of the 11+ charge state normalized to the phosphorylated form of P2Y_1_R. (ii) Upon addition of MRS2500, the population of free P2Y_1_R is reduced relative to the phosphorylated form (−21 ± 4%) and (iii) concomitant binding to the apo receptor is enhanced (17 ± 1.4%). (**B**) Schematic representation of ADP and drug binding to the phosphorylated receptor with phosphosites identified at the C terminus [serine residues (green)]. ADP binding takes place with equal probability to phosphorylated and nonphosphorylated forms. By contrast, drug binding occurs preferentially to the nonphosphorylated form.

Because we observed that the antagonist MRS2500 inhibits phosphorylation of P2Y_1_R, whereas binding of ADP/ATP does not, we set out to establish a link between drug binding and phosphorylation. We identified phosphosites in apo P2Y_1_R, without addition of drug, using conventional phosphoproteomics (table S2). Three phosphoserine residues were located at the C terminus: S346, S352, and S354. These three residues are not phosphorylated in the presence of MRS2500, implying that drug binding during preparation inhibits the phosphorylation reaction. If these three phosphorylated residues at the C terminus modulate the structure and dynamics of the GPCR, then we would anticipate a reduction in drug binding to the phosphorylated form of the protein due to restricted access to the binding site. To test this hypothesis, we incubated MRS2500 with a mixture of unmodified and the phosphorylated apo form of the protein. Even though the level of phosphorylation is quite low (sum of all three sites, <40%), we found that drug binding was predominantly to the nonphosphorylated form, with a reduced population (~20%) of MRS2500 bound to the phosphorylated receptor (Fig. 3).

Because the activation of GPCR kinases (GRKs) relies on direct docking with active GPCRs (*27*), we hypothesize that the restrained conformation of MRS2500-P2Y_1_R restricts access of GRKs and thereby inhibits receptor phosphorylation. This finding is consistent with recent molecular dynamics simulations, wherein an ionic lock was found to tether helices in the MRS2500-P2Y_1_R complex but was absent in the ADP-bound receptor (*28*). Because GPCR phosphorylation is considered a critical step that facilitates binding to interacting proteins such as arrestin (*29*, *30*), our observations implicate MRS2500 in modulating P2Y_1_R function not only through prevention of dynamics but also through inhibition of phosphorylation-dependent protein interactions. Moreover, preferential MRS2500 binding to the nonphosphorylated receptor highlights the possibility of an allosteric effect of C-terminal phosphorylation, hindering access to the drug-binding site. This phenomenon was also reported in the study of β2-adrenoceptor (*31*), and it raises the potential of GRKs to modulate the pharmacology of MRS2500.

## DISCUSSION

By developing and applying MS to retain ligand binding in a GPCR, we have demonstrated binding of the endogenous ligand ADP to both unmodified and phosphorylated forms of the receptor. We have also shown that high concentrations of ATP attenuate binding of ADP but do not completely displace ADP (fig. S2), in line with the known ability of ATP to act as a partial agonist/antagonist depending on the expression level of P2Y_1_R (*32*–*34*). By contrast, we have shown that ATP at the same levels can displace MRS2500 completely, which subsequently enables ADP binding to P2Y_1_R to trigger activation of the receptor. Our observation is supported by reports of a weak competition of MRS2500 by saturated 2-methyl-thio-ADP on the cell membranes of Sf9 insect cell overexpressing P2Y_1_R (*35*), and it provides a rationale for the moderate side effects reported during treatment with MRS2500 (*15*, *17*).

The possibility of an additional binding site for ATP in P2Y_1_R has also been raised (*36*), and although the presence of excess ATP used in our experiments means that we cannot discount nonspecific binding, our data are consistent with the existence of a second ATP-binding site. Of particular note in our study is the observation that the drug inhibits phosphorylation and binds preferentially to the unphosphorylated receptor. These observations highlight the significant advantages of MS over other biophysical approaches because the influence of PTMs can be related directly to drug binding.

Overall, given the well-documented challenges of isolating sufficient quantities of GPCRs, with folding and ligand binding abilities preserved, our study, which is relatively rapid (hours) and requires only tens of microliters at micromolar concentrations of protein, exemplifies a powerful new technology. Moreover, because GPCRs are the largest class of drug targets in the human genome, the molecular basis of their activation, the competitive binding of their inhibitors, and the impact of PTMs on downstream coupling and ligand binding provide invaluable information for drug development. This MS approach therefore represents a significant advance over many current screening assays and heralds new opportunities for targeted GPCR drug discovery.

## MATERIALS AND METHODS

### P2Y_1_R expression and purification

P2Y_1_R was overexpressed in insect cells, and the cell membranes were enriched as described previously (*18*). To purify P2Y_1_R without drug binding, the membranes were solubilized in 50 mM Hepes (pH 7.5), 300 mM NaCl, 0.5% (w/v) DDM (Anatrace), and 0.1% (w/v) cholesteryl hemisuccinate (CHS) (Sigma) for 3 hours at 4°C. The debris was pelleted by centrifugation at 160,000*g* for 30 min, and the supernatant was collected with supplement of imidazole (pH 7.5) to a final concentration of 30 mM. After incubation of supernatant with TALON immobilized metal affinity chromatography resin (Clontech) overnight at 4°C, the resin was harvested and first washed with 10 column volumes of 25 mM Hepes (pH 7.5), 300 mM NaCl, 10% (v/v) glycerol, 40 mM imidazole, 0.05% (w/v) DDM, and 0.01% (w/v) CHS, followed by extensive washing with 10 column volumes of 25 mM Hepes (pH 7.5), 300 mM NaCl, 10% (v/v) glycerol, 0.05% (w/v) DDM, 0.01% (w/v) CHS, 10 mM MgCl_2_, and 5 mM ATP and 15 column volumes of 25 mM Hepes (pH 7.5), 300 mM NaCl, 10% (v/v) glycerol, 0.05% (w/v) DDM, and 0.01% (w/v) CHS. The protein was eluted by 25 mM Hepes (pH 7.5), 300 mM NaCl, 10% (v/v) glycerol, 300 mM imidazole, 0.05% (w/v) DDM, and 0.01% (w/v) CHS, followed by removing imidazole with PD MiniTrap G-25 column (GE Healthcare). The C-terminal His-tag and glycosylation of receptor were further removed by treating PreScission protease (in-house) and peptide *N*-glycosidase F (in-house) overnight. The purification of MRS2500-bound receptor followed the published protocol (*1*).

### Nondenatured MS for intact P2Y_1_R

Apo and drug-bound P2Y_1_R were buffer-exchanged into 200 mM ammonium acetate containing DDM, foscholine, and CHS and immediately introduced into a modified Q Exactive mass spectrometer according to a previously reported method (*20*, *37*). Briefly, a gentle voltage gradient was applied (injection flatapole, inter-flatapole, bent flatapole, and transfer multipole: 7.9, 6.94, 5.9, and 4 V, respectively) to avoid the collisional activation of the ions before transferring into the higher-energy collisional dissociation (HCD) cell. The optimized acceleration voltage (150 V) was then applied to the HCD cell to remove the detergent micelle from the protein ions. Spectra were acquired with 10 microscans and averaged with a noise level parameter of 3. Backing pressure was maintained at ~1.05 × 10^−9^ mbar to allow better transmission of protein ions. Data were analyzed with Xcalibur 2.2 SP1.48.

### Proteomic analysis of P2Y_1_R

P2Y_1_R in protein purification buffer was digested with trypsin through filter-aided sample preparation (*38*, *39*). Protein was denatured in 100 mM ammonium bicarbonate buffer containing 8 M urea and 1% sodium deoxycholate and transferred to Microcon YM-30 (Millipore) for further reduction and alkylation by tris-(2-carboxyethyl)phosphine and chloroacetamide. The tryptic peptides were harvested by centrifugation after digestion. For MS analysis, peptides were separated on an Ultimate 3000 UHPLC system (Thermo Fisher Scientific) and electrosprayed directly into a Q Exactive mass spectrometer (Thermo Fisher Scientific) through an EASY-Spray nanoelectrospray ion source (Thermo Fisher Scientific). The peptides were trapped on a C18 PepMap100 precolumn (300-μm inside diameter × 5 mm, 100 Å; Thermo Fisher Scientific) using solvent A (0.1% formic acid in water) at a pressure of 500 bar. The peptides were separated on a C18 PepMap RSLC Nano Easy column (2 μm, 100 Å; Thermo Fisher Scientific) using a linear gradient [length, 30 min; 7 to 28% solvent B (0.1% formic acid in acetonitrile); flow rate, 200 nl/min]. The raw data were acquired on the mass spectrometer in a data-dependent mode. Full-scan spectra were acquired in the Orbitrap [scan range, 350 to 2000 *m*/*z*; resolution, 70,000; automatic gain control (AGC) target, 3 × 10^6^; maximum injection time, 50 ms]. After the MS scans, the 20 most intense peaks were selected for HCD fragmentation at 30% of normalized collision energy. HCD spectra were also acquired in the Orbitrap (resolution, 17,500; AGC target, 5 × 10^4^; maximum injection time, 120 ms), with first fixed mass at 180 *m*/*z*.

### Phosphosite identification of P2Y_1_R

Raw MS data were processed by MaxQuant (version 1.5.0.35) for peak detection and quantification. MS spectra were searched against the UniProt Homo sapiens database (version 2013/04/03) as well as a list of common contaminants using the Andromeda search engine (*40*, *41*) with the following search parameters: full tryptic or chymotryptic specificity, allowing two missed cleavage sites; fixed modification was set to carbamidomethyl (C); and the variable modification was set to acetylation (protein N terminus) and oxidation (M). Mass spectra were recalibrated within MaxQuant with a precursor error tolerance of 20 parts per million (ppm) and then re-searched with a mass tolerance of 5 ppm. The search results were filtered with a false discovery rate of 0.01 for proteins, peptides, and peptide spectra matches.

## Supplementary Material

http://advances.sciencemag.org/cgi/content/full/3/6/e1701016/DC1

## SUPPLEMENTARY MATERIALS

Supplementary material for this article is available at http://advances.sciencemag.org/cgi/content/full/3/6/e1701016/DC1 fig. S1. Dissociation of the P2Y_1_R-MRS2500 complex in the gas phase. fig. S2. Mass spectrum of wild-type P2Y_1_R incubated with different molar ratios of ATP. table S1. Measured and calculated mass differences of P2Y_1_R in apo and ligand-bound forms. table S2. P2Y_1_R phosphopeptides identified by liquid chromatography–MS/MS analysis.
