# Supplementary material for: Ligand binding to a G protein–coupled receptor captured in a mass spectrometer
Source: Sci Adv. 2017 Jun 16;3(6):e1701016. doi: 10.1126/sciadv.1701016 (PMC5473672; doi:10.1126/sciadv.1701016)
Supplement: http://advances.sciencemag.org/cgi/content/full/3/6/e1701016/DC1 [file supp_3_6_e1701016__index.html]

Science Advances | Science Advances

## Supplementary Materials

**This PDF file includes:**

- fig. S1. Dissociation of the P2Y1R-MRS2500 complex in the gas phase.
- fig. S2. Mass spectrum of wild-type P2Y1R incubated with different molar ratios of ATP.
- table S1. Measured and calculated mass differences of P2Y1R in apo and ligand-bound forms.
- table S2. P2Y1R phosphopeptides identified by liquid chromatography–MS/MS analysis.

Download PDF

**Files in this Data Supplement:**

- Adobe PDF - 1701016\_SM.pdf
