# Supplementary material for: Ligand binding to a G protein–coupled receptor captured in a mass spectrometer
Source: Sci Adv. 2017 Jun 16;3(6):e1701016. doi: 10.1126/sciadv.1701016 (PMC5473672; doi:10.1126/sciadv.1701016)
Supplement: http://advances.sciencemag.org/cgi/content/full/3/6/e1701016/DC1 [file 1701016_SM.pdf]

Science Advances

AAAS

advances.sciencemag.org/cgi/content/full/3/6/e1701016/DC1

Supplementary Materials for

Ligand binding to a G protein–coupled receptor captured in a mass spectrometer

Hsin-Yung Yen, Jonathan T. S. Hopper, Idlir Liko, Timothy M. Allison, Ya Zhu, Dejian Wang, Monika Stegmann, Shabaz Mohammed, Beili Wu, Carol V. Robinson

Published 16 June 2017, *Sci. Adv.* **3**, e1701016 (2017)
DOI: 10.1126/sciadv.1701016

This PDF file includes:

- fig. S1. Dissociation of the $P2Y_1R$-MRS2500 complex in the gas phase.
- fig. S2. Mass spectrum of wild-type $P2Y_1R$ incubated with different molar ratios of ATP.
- table S1. Measured and calculated mass differences of $P2Y_1R$ in apo and ligand-bound forms.
- table S2. $P2Y_1R$ phosphopeptides identified by liquid chromatography–MS/MS analysis.

Supplementary Materials

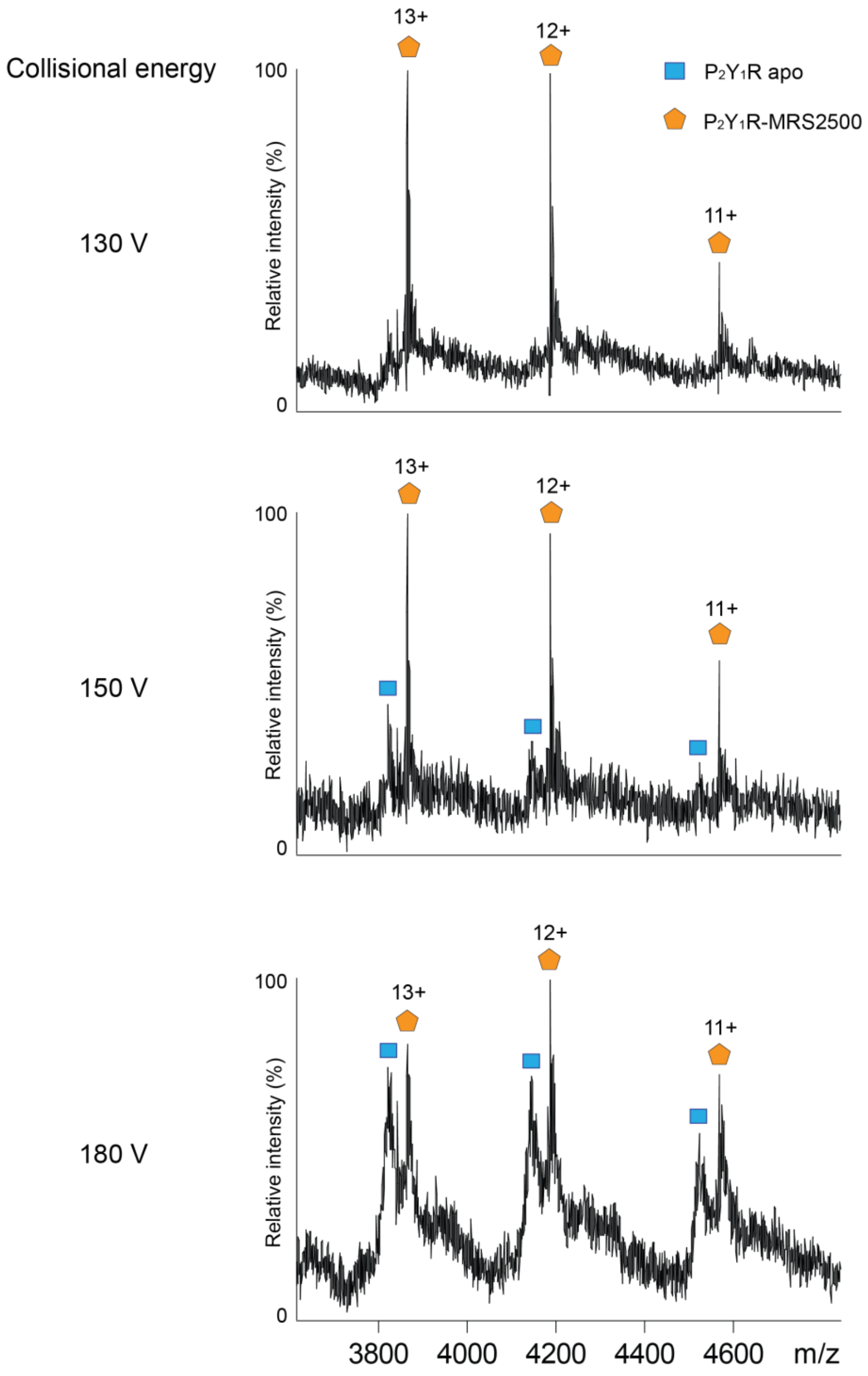

fig. S1. Dissociation of the P2Y1R-MRS2500 complex in the gas phase. The spectrum of P2Y1R co-purified with MRS2500 reveals 100% binding MRS2500 to the receptor (Upper spectrum). Increase of the collision energy applied in the mass spectrometer induces dissociation of MRS2500 from P2Y1R, confirming the non-covalent drug-binding of P2Y1R (Middle and bottom spectra).

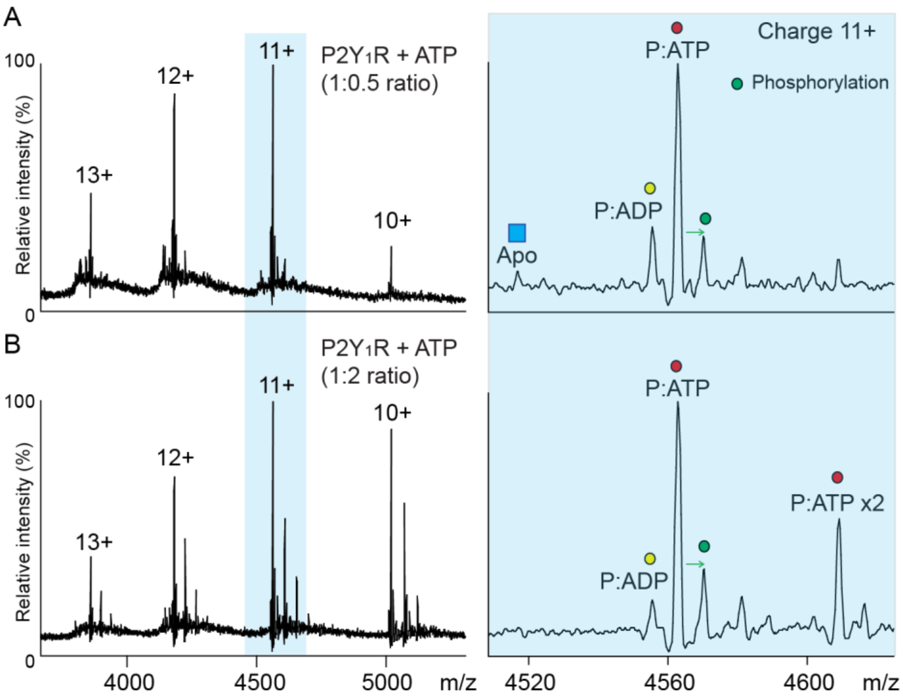

fig. S2. Mass spectrum of wild-type P2Y$_1$R incubated with different molar ratios of ATP.
(**A**) P2Y$_1$R incubated with ATP at a 1: 0.5 molar ratio shows displacement of ADP by ATP and (**B**) P2Y$_1$R with ATP at a 1: 2 molar ratio shows further displacement of ADP and also possible evidence of a second binding site for ATP.

table S1. Measured and calculated mass differences of $P2Y_1R$ in apo and ligand-bound forms.

	Measured mass (Da)	Mass difference to apo measured (Da)	Mass difference to apo calculated (Da)
Apo $P2Y_1R$	49685.1 ± 0.7		
$P2Y_1R$ +ADP	50111.8 ± 1.3	426.7 ± 2.0	427.2
$P2Y_1R$ +ATP	50191.3 ± 1.2	506.2 ± 1.9	507.2
$P2Y_1R$ + MRS2500	50247.7 ± 1.0	562.6 ± 1.7	561.3

1. Calculated mass of $P2Y_1R$ according to protein sequence is 49556.7 Da
2. Receptor possesses 2 conserved disulfide bonds between Cys124-202 and Cys 42-296 to reduce calculated mass to 49552.7 Da .
3. The mass increase of apo $P2Y_1R$ determined experimentally is 132.4 Da, which contains carboxyamidomethylation on two cysteins, oxidation and deglycosylation occurred during purification.

table S2. $P2Y_1R$ phosphopeptides identified by liquid chromatography–MS/MS analysis.

Peptide sequence	Phosphosites	Miss cleavage	z	*m/z*
ASRRp**S**EANLQSK	S346	2	2	672.85
ASRRp**S**EANLQSK	S346	2	3	448.57
RSEANLQp**S**Kp**S**EDMTLNILPEFK	S352/S354	2	2	1274.64
RSEANLQp**S**Kp**S**EDMTLNILPEFK	S352/S354	2	3	849.76
RSEANLQp**S**Kp**S**EDMTLNILPEFK	S352/S354	2	4	637.32
